# A Scale for the Management of Aggressive and Violent Behaviour (C_MAVAS): Psychometric Properties Testing in Mental Health Nurses

**DOI:** 10.3390/ijerph15071496

**Published:** 2018-07-16

**Authors:** Teris Cheung, Jolene Mui, Yuen Shan Ho, Wai Tong Chien

**Affiliations:** 1 School of Nursing, Hong Kong Polytechnic University, Hong Kong, China; janice.ys.ho@polyu.edu.hk; 2 Castle Peak Hospital, Hospital Authority, Hong Kong, China; muihc@ha.org.hk; 3 Nethersole School of Nursing, Chinese University of Hong Kong, China; wtchien@cuhk.edu.hk

**Keywords:** MAVAS, psychometric properties, aggression, violence, mental health nurses, psychiatric hospitals, exploratory factor analysis

## Abstract

*Background*: This study set out to examine the psychometric properties of C_MAVAS, a newly Chinese-translated version of MAVAS, a 27-item scale assessing healthcare professionals’ attitudes to the management of patient violence. *Method*: The English version of the MAVAS was translated and back-translated to come up with C_MAVAS. A convenience sample of 262 qualified mental health nurses working in a local psychiatric hospital was recruited. Exploratory factor analysis tested C_MAVAS’s construct validity. *Results*: Content validity of the C_MAVAS was very satisfactory with validity indices of 97.4% for the overall scale and 90% to 100% for individual items. Exploratory factor analysis yielded a four-factor solution: ‘interactional perspectives on patient violence’, ‘best ways perceived for violence management’, ‘internal or biomedical perspectives on patient violence’, and ‘external perspectives on patient aggression and violence’, were important in shaping their attitudes towards managing violence and patient disruptiveness. Internal consistency of the Chinese version was barely satisfactory (Cronbach’s alpha = 0.51–0.67) for the four factors/subscales and its test-retest reliability was good (Pearson’s coefficient = 0.84). *Conclusion*: The findings suggest the C_MAVAS is a valid and reliable tool to measure mental health nurses’ attitudes towards patient violence/aggression in a mental hospital setting.

## 1. Introduction

Workplace violence is a significant problem in China and internationally. In 2013, 25,630 incidents of workplace violence (WPV) were reported in the US, 74% of which occurred in healthcare settings [1]. Research has consistently reported that the rates of violence in public healthcare settings exceed those in private medicine. The threat of violence affects both private- and public-sector healthcare workers across America [2].

In Asia, a number of cross-sectional studies have examined the prevalence of WPV over the last five years. Studies showed that nurses, in particular, were exposed to WPV at an alarming degree. In 2017, 44.6% (*n* = 850) of a cohort of nurses had experienced some form of WPV in the preceding year [3]. The most common perpetrators of WPV were patients (36.6%) and their relatives (17.5%), followed by colleagues (7.7%) and supervisors (6.3%). Another similar study [4] found that 81.5% of nurses (*n* = 613) and 14.9% of physicians (*n* = 107) in Macau had suffered WPV in the preceding year. The most common forms of workplace violence were verbal abuse (53.4%), physical assault (16.1%), bullying or harassment (14.2%), sexual harassment (4.6%), and racial harassment (2.6%). Another Macau-based study reported comparable levels of prevalence of verbal abuse (53.4%), physical assault (16.1%), bullying (14.2%), and sexual harassment (4.6%) towards 412 physicians and nurses [5] (*n* = 720). Mai’s study, though, did not separate out the prevalence rates of violence towards doctors and nurses. Another cross-sectional survey conducted in Heilongjiang, China examined the prevalence of WPV on 886 nurses, of which 67.2% reported different forms of WPV in the past 16 months [6].

Compared with workers in other fields, medical and healthcare professionals have a greater likelihood of being attacked by patients, and the risk for facing violence among clinical nurses are even higher than other medical and allied health personnel [7]. Nurses are arguably the most at-risk group among all the professionals who have to manage patients’ aggression or violence [3,4,6,8,9,10]. Psychiatric settings are one of the most frequent health care settings in which violence occurs [11] and mental health nurses are the most vulnerable group of WPV in psychiatric settings [12]. However, very few studies over the last decade have examined nurses’ attitudes towards the violence encountered from patients, especially psychiatric in-patients [11,12]. Nonetheless, these attitudes on the nurses’ part remain a critical factor affecting their clinical judgment and care of their patients in healthcare settings [13,14]. It has been shown that nurses viewing violence “positively” (or tolerantly) are more likely to manage it with interpersonal approaches [13], while nurses with negative attitudes may attempt to use coercive measures, sometimes involving unnecessary physical and chemical restraints [15]. Therefore, understanding nurses’ attitudes towards patient aggression/violence becomes vitally important in seeking to formulate an effective approach to managing their disruptiveness in psychiatric care settings.

There are a few instruments that are commonly used to measure nurses’ attitudes, including the Attitudes Toward Patient Physical Assault Questionnaire (ATPPAQ) [16], the Attitude Toward Aggressive Behaviour Questionnaire (ATABQ) [17], the Perception of Aggression Scale (POAS) [18]; the Attitude Toward Aggression Scale (ATAS) [19], and the Management of Aggression and Violence Attitude Scale (MAVAS) [14]. 

The ATPPAQ is a self-report questionnaire comprising 31 statements ranged on a five-point Likert scale (“strongly disagree” to “strongly agree”). The questionnaire addresses four domains: (1) staff beliefs and concerns about their safety; (2) staff competence; (3) staff performance; and (4) legal issues, e.g., concerning patient responsibility for their behaviour. The ATPPAQ exhibits satisfactory test-retest reliability (*r* = 0.69) [16]. The ATABQ [17] consists of 12 statements again adjudged by a five-point Likert Scale (from “strongly agree” to “strongly disagree”). It was explicitly designed for evaluating an intervention on prevention and management of patient aggressiveness. Its reliability (Cronbach’s alpha = 0.69) comes out as reasonable and its test-retest reliability as top-notch (*r* = 0.972).

Of these psychological instruments, the POAS and MAVAS seem the most widely used internationally in healthcare studies. The English version of the POAS, however, has only recently been translated and validated in Hong Kong [12]. The MAVAS is used in psychiatric care worldwide; and its transferability has been established in a European mental health inpatient setting [20]. Some researchers [11] have further tested the psychometric properties of the MAVAS using the 23-item MAVAS-BR in Brazil. DeVargas et al.’s study [11] concluded the MAVAS-BR represented a valid, reliable scale for gauging nurses’ attitudes towards patient aggression. Another relevant recent study investigated MAVAS’s psychometric properties in an emergency care setting in Hong Kong [12]; the researchers replicated de Vargas’s finding that the MAVAS was a valid and reliable assessment tool in representing emergency nurses’ attitudes towards its theme. Wong and Chien’s study [12] used the original English version of the MAVAS, opening the possibility of their misreporting nurses’ attitudes, on the basis that most nurses in Hong Kong are Chinese-speakers. Given the fact that WPV remains prevalent in Hong Kong [3] and other Chinese healthcare settings [4,9], it could be thought appropriate to validate a Chinese version of MAVAS in order to more accurately assess professionals’ attitude towards WPV. The lack of a Chinese-version of the MAVAS can serve as bridging the research gap in the measurement and understanding of professionals’ attitudes towards violent behaviour. This study was set out to do this, working primarily with mental health nurses working with inpatients in the psychiatric unit of a local hospital.

## 2. Materials and Methods 

### 2.1. Study Design and Translation Procedures

This study aimed at testing the psychometric properties of the translated Chinese version of the Management of Aggression and Violence Attitude Scale (C_MAVAS). The study relied on Brislin’s model of translation [21], involving three phases and four steps:

#### 2.1.1. Forward Translation

In step 1, a bilingual health professional with a mental-health background forward-translated MAVAS from English into the target language, Chinese. The translator both understood MAVAS and the target population and could be expected to maximize the equivalence of the translation and the currency, and appropriateness, of the terms in the Chinese text.

In step 2, a monolingual reviewer checked C_MAVAS for incomprehensible or ambiguous phrases. This reviewer did not have any knowledge of the original English version, making her more likely to capture grammatical errors and lapses of idioms in the translation.

#### 2.1.2. Backward Translation

In step 3, C_MAVAS was back-translated by another bilingual researcher, who was ‘blinded’ to the original English. This method facilitated a faithful rendering of the Chinese into the original source-language [21]. Step 4 involved the principal investigator comparing the back-translated version of C_MAVAS with the original for linguistic congruence and cultural sensitivity. Items showing discrepancies between the original and back-translated English were referred back to the translators iteratively until maximum equivalence, and full agreement, was attained among translators and between the source language and the back-translated versions of the scale.

### 2.2. Psychometric Testing

#### Content Validation

An expert panel comprising 10 mental health professionals (two psychiatrists, one clinical psychologist, three mental health nursing academics, two advanced practice or consultant psychiatric nurses, and two nursing managers) evaluated C_MAVAS’s content validity. Panel members were apprised of the study’s aims and key definitions, including its procedure of rating attitudes on a five-point scale. Each panel member was asked to rate each of the 27 items on a four-point scale (1: ‘irrelevant’; 2: ‘items need major revision to be relevant’; 3: ‘items need minor revision to be relevant’; and 4: ‘highly relevant’). Content validity for the C_MAVAS was assessed through a calculation of the Content Validity Index (CVI) synthesising expert ratings. Item-level CVIs were calculated by the proportion of panel experts rated an item ‘3’ or ‘4’.

The expert panel duly confirmed C_MAVAS’s face validity. Two panel experts raised concerns over one item, “All patients are verbally abusive”, which they considered an over-statement, suggesting a revision to “Patients may be verbally abusive”. Experts further found “Other people make patients aggressive or violent” unclear, recommending a specification of ‘other people’ (e.g., other patients, medical-related personnel, family members, or strangers etc.). The justification for these changes was that the first statement measured nurses’ beliefs concerning the clinical management of patient aggression (Theme 4), while the other solicited views regarding interactions and personal relationships between nurses and patients (Theme 1). These explanations obviated any other amendments for the sake of face validity. Likewise, all panelists described the C_MAVAS as clear, including in its instructions, and easy to read. 

Descriptive analysis and inferential statistics analysed the data using SPSS version 24.0 (SPSS Inc., Chicago, IL, USA). The minimum level of agreement among 10 panelists was set at ≥0.62, at a 0.05 level of significance [22]. In other words, seven out of ten experts had to agree on content validity for an item to make it to the final instrument. Item- and scale-level CVIs of >0.8, using the averaging method, were required. For scale-level content validity (S-CVI), the S-CVI/Average measured the average proportion of items judged relevant by the 10 experts, or, in other words, the average level for the I-CVI across items. This S-CVI/Average had to be 0.9 for the scale to pass this content validity testing [23].

In our study, the I-CVIs for 20 items are 1.0 and for the remaining seven items 0.9. The S-CVI/Ave (Scale-CVI, Averaging method) scores 0.974. This implies that all 27 items were agreed on by experts as relevant to their thematic domains.

### 2.3. Ethical Considerations

Ethical approval (reference no: HSEARS20170512001) was obtained from the Clinical Research Ethics Committees, the New Territories West Cluster of Hospital Authority, Hong Kong and the Human Subjects Research Ethics Subcommittee of the Hong Kong Polytechnic University. Participants were duly informed of the study’s purpose and assured of the confidentiality and security of their data. The questionnaire’s return was taken as participants’ informed consent. All questionnaires were anonymously coded by the researcher before distribution. Participants could withdraw from the study without any negative consequence. All data was stored in a locked cabinet with access restricted to the research team only. 

### 2.4. Data Collection

#### 2.4.1. Participants and Study Setting

A convenience sample of 40 bilingual registered psychiatric nurses working in a regional psychiatric hospital was asked to complete MAVAS (in English) and C_MAVAS. Half the nurses (*n* = 20) were administered the C_MAVAS, followed by the original English version, while the other half worked through the two versions in a reversed order. This split-half technique avoided triggering respondents’ recall bias [24]. 

Next, a convenience sample of 324 qualified nurses working in a large-scale regional mental hospital was approached to test the new scale’s construct validity and test-retest reliability [25]. As at least 10 participants were required per item [26], this sample size was adequate for exploratory factor analysis after considering for a 20% non-response rate. Each participant was invited to fill in the C_MAVAS and a socio-demographic sheet. Half of them (*n* = 162) filled in C_MAVAS again at a four-week interval for test-retest reliability. All the test-retest surveys were assigned an identical code by the researcher.

#### 2.4.2. Inclusion and Exclusion Criteria

To be included in the sample, subjects had to be: (a) front-line psychiatric nurses; (b) aged between 21 and 60; (c) working full-time (44 hours/week) in the hospital under study; (d) registered or enrolled mental health nurses with the Hong Kong Nursing Council; and (e) proficient in reading Chinese and English languages. Part-time nurses or those without direct patient-care duties were excluded.

The C_MAVAS was distributed to different clinical units with the assistance of the study hospital’s Central Nursing Division. The scale, together with information about the purpose and procedure of the study and contact information of the principal investigator, was enclosed in a self-addressed envelope. Participants assigned to undertake test-retest reliability picked up two C_MAVAS and two self-addressed envelopes. Participants returned their completed questionnaires in a sealed collection box at the hospital’s Central Nursing Division (CND). A gentle reminder was issued twice to all eligible respondents (at two and four weeks after) to boost the response rate.

#### 2.4.3. Measures

The original English version of the Management of Aggression and Violence Attitude Scale (MAVAS) was developed by [14]. MAVAS examine nurses’ (or, alternatively, patients’) attitudes towards the causes of patients’ aggression and towards methods of dealing with this disruptiveness. MAVAS contains 27 statements, 13 of which deal with what motivates aggressiveness, and 14 violence management [14]. Duxbury’s scale [14] solicits views relating to four factors, ‘interactional perspectives on patient aggression and violence’; ‘external perspective on patient aggression and violence’; ‘internal (biomedical) perspectives on patient aggression and violence’; and ’views about (the) manage (ment of) patient aggression or violence’. MAVAS has demonstrated good test-retest reliability (Pearson’s *r* = 0.89 & 0.85) [12,20] in the UK and Hong Kong respectively.

We also adopted the original MAVAS’s interpretation of scored responses using a five-point Likert scale (1–5), where 1 indicates ‘strongly agree’ and 5 ‘strongly disagree’. Lower scores denote higher levels of agreement with given statements. 

The study collected socio-demographic data from respondents for their gender, age, marital status, years of post-registration experience, clinical specialty, and frequency of violence/aggressive episodes they had encountered in the past 12 months. Participants were also asked if they had sustained any injury and/or reported the incident(s); whether they had received violence management training in the past five years; and whether they felt safe at work (yes/no).

## 3. Data Analysis

Responses to the 27 scale items were entered into SPSS Version 24.0. Non-entered scale items were replaced by item means [26], unless 30% (i.e., 9) items or above had been missed, in which case responses were excluded from the data analysis. 

As the factor structure of this instrument was not well-established and similar across cultures, we decided to use confirmatory factor analysis (CFA) to validate the construct validity. The CFA model with four factor structures suggested by the original authors [20] was conducted. Three goodness-of-fitness indices were used to assess the model fitness include comparative fit index (CFI), Tucker-Lewis index (TLI), and root mean square error of approximation (RMSEA). According to Hu and Bentler [27], both CFI and TLI values of greater 0.9 suggested an acceptable model for the data. For the RMSEA, the values were <0.05 indicates good fit (i.e., model fit values between 0.05 and 0.08 are acceptable; and those > 0.10 suggest poor model fit). However, we had a poor goodness-of-fit index for the model, which had the values of 0.563 and 0.517 for CFI and TLI, respectively, and RMSEA was 0.068. The same situation was reported in the validation of the same scale (MAVAS) in the Brazil version [28]. We then followed the same strategy and employed the exploratory factor analysis to find the factors.

### Exploratory Factor Analysis

Exploratory factor analysis (EFA) was then employed to establish C_MAVAS’s construct validity. Before running the factor analysis, the factorability of the C_MAVAS data was examined using Bartlett’s test of sphericity (*p* < 0.05) and the Kaiser-Meyer-Olkin (KMO) test (index value of KMO > 0.6). The eigenvalues of C_MAVAS’s factors were examined by a scree plot in which the shape of the curve was expected to change direction and flatten. All factors above the elbow, or break in the plot, would contribute to the largest proportion of the total variance of the construct for “attitude to violence” in the dataset. Varimax rotation elicited the significant domains or dimensions of the C_MAVAS along a principal axis. Factors emerging with eigenvalues > 1 were considered as potentially meaningful in determining nurses’ attitudes towards patient aggression and violence. We followed the same validation criteria as the MAVAS in its first formulation [11,14,20] in which items with a factor loading of ≥0.30 were kept on the scale. Cronbach’s alpha coefficient assessed the internal consistency of the C_MAVAS overall scale and of its subscales. Values between 0.7 and 0.9 indicated high reliability, and between 0.5 and 0.7 moderate reliability [28,29]. Test-retest reliability over a four-week interval was measured using an intra-class correlation coefficient (ICC). ICC values >0.9 indicated very satisfactory test-retest stability, and values between 0.75 and 0.90 indicated moderate test-retest reliability.

## 4. Results

### 4.1. Participants’ Sociodemographic Characteristics

A total of 263 nurses returned their questionnaires. However, one questionnaire with nine items missing on the C_MAVAS was excluded from analysis. The 262 participants of our study had a response rate of 80.9%; of these, 98 participants completed the test-retest surveys (42.2%). Of respondents, 61.1% were female and married (64.4%); and about three-quarters aged between 30 and 55 (74.3%), with at least an undergraduate education (73.6%). Seventy percent were registered or enrolled psychiatric nurses, 30% were advanced practice nurses or above, 81% had fewer than 15 years of working experience in a psychiatric hospital, and 80.2% had encountered some degree of violence or aggression from patients in the past 12 months. The most common types of violence reported by respondents were verbal abuse (79.3%) and physical assault (66.1%). Most respondents (96.6%) had attended training course(s) on dealing with patient violence in the past five years. A significant fraction of respondents (77.1%) reported thought that violent incidents could be prevented; and 86.6% found their workplaces safe. Table 1 summarises the 262 respondents’ demographic and occupational-related characteristics. Table 2 summarises the 262 respondents’ experience of WPV in the past 12 months.

### 4.2. Construct Validity

Construct validity of the C_MAVAS was assessed using exploratory (principal component) factor analysis (EFA). A Kaiser-Meyer-Olkin (KMO) test yielded a value of 0.693 (i.e., >0.60 as recommended), while Barlett’s test of sphericity was <0.001, indicating adequate factorability of C_MAVAS for the EFA. From the un-rotated matrix of the EFA, results revealed four components with eigenvalues > 1.6. An inspection of the scree plot revealed a clear break after the fourth factor, meaning the four-factor solution was retained for factor rotation (Table 3).

The results of Varimax rotation indicated that 26 out of 27 items had a factor loading of >0.30 on the four factors, explaining 36.0% of the total variance (Table 3). There was one item (“Patients who are aggressive towards staff should try to control their feelings”) found to overlap between two factors (1 and 4) after rotation (item factor loadings were 0.313 and 0.338). This item was then allocated onto the factor with the higher loading and found closer to other items in that grouping. After careful examination of all items’ contextual meaning and a review of the relevant literature, these four factors were renamed as F1—interactional perspectives on patient aggression and violence, F2—best ways perceived for violence management, F3—internal or biomedical perspectives on patient violence, and F4—external perspectives on patient aggression and violence. One item, “Patients are aggressive because they are ill”, did not fit with any of the four factors.

### 4.3. Internal Consistency and Test-Retest Reliability

The Cronbach’s alpha coefficient of the overall C_MAVAS came in at 0.744. The alpha coefficients of the four subscales (factors) were 0.672 (Factor 1), 0.649 (Factor 2), 0.634 (Factor 3), and 0.510 (Factor 4). If the item “Patients are aggressive because they are ill.” was deleted, the alpha coefficient of the overall scale was decreased to 0.739. Test-retest reliability over a four-week interval was satisfactory, with the intra-class correlation coefficient (ICC) of 0.837 for overall scale.

## 5. Discussion

We recruited 262 participants in this study and this sample was lower than our sample size estimation (*n* = 324), nevertheless, the overall response rate was very high. The completion of test-retest surveys was also satisfactory. The total number of participants was sufficient to examine C_MAVAS’s psychometric properties [26]. Our results from the KMO test and Barlett’s Test of Sphericity supported the factorability of the dataset.

Test-retest reliability of the C_MAVAS indicated consistent responses across all items over a four-week interval. Our result is very close to a similar study conducted by Wong and Chien [12] (ICC = 0.85) in Hong Kong and comparable to the original scale developed by [14] (ICC = 0.89). Exploratory factor analysis revealed that nurses’ item-responses to the C_MAVAS could be explained with reference to a four-factor structure. Our factor structure bore close comparison to the original MAVAS [14]—possibly since it was also conducted in a psychiatric hospital, albeit in a different cultural context. 

The Cronbach alpha of Factor 4 “external perspectives on patient aggression and violence” is 0.51, which is considered to be of moderate reliability according to Hinton et al. [28]. The reliability of the full scale in this study is 0.744, which is quite similar with that of the full scale of the Brazil version (0.75), and can be considered satisfactory. When considering the reliability of the scale, it should be noted that its reliability of the scale is confirmed with its stability in the course of time (repeatability); its language modifications needs further testing in the specific context/setting, as their reliability in terms of internal consistency is insufficient [30]. Indeed, the test-retest reliability (0.837) in our study showed that reliability is satisfactory.

Although the explained variance of 36.0% is considered to be low, it was observed that all of the factors evaluated had statistically significant correlations with the full scale of MAVAS; the correlation coefficients ranged between *r* = 0.421 (*p* < 0.01) and *r* = 0.541 (*p* < 0.01). The highest correlation coefficient (*r* = 0.541) was observed between Factor 3, “Internal or biomedical perspectives on patient violence”, and the full version of MAVAS, and the lowest coefficient of correlation was observed between Factor 4, ‘external perspectives on patient aggregation and violence” and the full version (*r* = 0.421). In the MAVAS Brazil version, the correlations of full scale and the individual factors ranged between 0.32 and 0.70. Since the factors include several items with a loading of 0.3, it is expected that the percentage of the variance explained will be reduced. Hair et al. [31] stated that a 0.30 loading translates to an approximately 10% variance explanation, and a 0.50 loading denotes that 25% of the variance is accounted for by the factor.

We found seven statements were associated with Factor 1; seven statements with Factor 2; four with Factor 3; and eight with Factor 4. Nurses in this study apparently attributed patient aggression to interactional factors and clinically managed these outbursts accordingly. They tended to rationalize patient violence by considering external factors (e.g. overcrowding; noisy clinical environments; rigid, highly structured routines, and stringent rules governing patients’ daily activities). Participants seemed to value the importance of establishing a therapeutic environment for patients in which they could communicate with staff as easily as possible. De-escalation skills and other clinical management approaches may effectively reduce the incidence of aggression and violence in clinical psychiatry. Internal factors (e.g., those pertaining to psychiatric diagnoses and patient characteristics) seems to be less influential in shaping nurses’ attitudes to patients’ aggressiveness. It is noteworthy that psychiatric nurses in the hospital should have attended a mandatory training course in the management of violence (Level 1 and 2) (de-escalation skills and breakaway techniques) before starting their jobs. These nurses should arguably be equipped with the pre-requisite theoretical and practical de-escalation skills to deal with patient violence [32]. The training sessions may also contribute to staff attitudes towards workplace violence.

Duxbury’s study [14] looked at both nurses’ and patients’ views regarding patient aggression and violence. This study restricted itself to the nurses’ views on the topic. Even though the factor structure of our scale is like that of the original MAVAS, there is still a need to consider variations in a cultural context. The study may have been subject to further sampling bias in that nurses who had dealt with patient aggression or violence may have been more likely to respond. The study participants may also have drawn disproportionately from the segment of potential respondents who had successfully dealt with patient aggression in the preceding year. These respondents may then have been more positively inclined to patient aggression and violence. A retrospective cross-sectional self-report design may also induce recall bias. We cannot, therefore, be confident that this study’s findings can be generalized to other settings.

Our factor structure differed in one important aspect from that identified by Wong and Chien [33]. Nurses in their study, conducted in an ER, were exposed to a higher risk of patient aggression than had they been working in a general setting [32,34]. Patients’ anger and upset, staff shortages, confusion between patients and staff, long waiting times, and delayed treatment may have tested patients’ tolerance. This would have spilled out in aggression [4]. It can be argued that physical settings, patient characteristics, and the staff-patient ratio all significantly affect nurses’ views regarding patient aggression. Thus, the variations in the factor structure between our study and Wong and Chien’s [12] may possibly be attributed by the nature of the healthcare settings. Future replication of this study may consider incorporating both nurses’ and patients’ views towards aggression/violence in general and psychiatric settings in Hong Kong. Findings may rule out the variations between the indifference of attitude toward patient aggression/violence between general and mental health nurses in different healthcare settings. 

The internal consistency of the original scale is not reported [14], though our overall Cronbach’s alpha coefficient was 0.74. The four factor solutions explained 36.0% of the total variance of nurses’ attitudes towards patient aggression, ranging from 6.84% to 14.87% per factor. A desirable level of explanation for variance for factor solutions has been pitched at 50% [35]; other researchers have held a minimum explanation of variance of 30% as sufficient [11]. We can possibly explain the lower total variance in our study by noting that three C_MAVAS items asked about secluding patients. In fact, seclusion is no longer a common practice in psychiatric settings in Hong Kong, partly due to space constraints. Another three items asked about the effect of the physical environment in relation to patient aggression. Our research hospital had different sorts of clinical wards (e.g., gazetted; non-gazetted, including long-stay wards; rehabilitation wards; specialty wards, such as for children and adolescents; psychogeriatric; intellectual disability; and forensic units). Patient characteristics may be a significant variable influencing nurses’ perspectives on patient violence and aggression. Two items asked about the use of medication. The original MAVAS was developed over fifteen years ago, meaning that medical advances in the formulation of a second generation of psychotropic drugs may effectively reduce patients’ psychotic symptoms, which may, in turn, lead to them losing control and giving way to violence in clinical settings less frequently. Therefore, we should be careful not to suppose too readily that C_MAVAS replicates MAVAS in these items.

Although the same four-factor structure appears to attach to this scale as to the original MAVAS, the themes and content of some single items and subscale items may not be applicable to psychiatric settings in Hong Kong. To better understand nurses’ views about patient aggression and violence, we suggest that replication of this study should abbreviate C_MAVAS by combining similar themes and dropping mention of seclusion. It would be interesting to incorporate patients’ views towards patient aggression to see if these converge with, or diverge from, those of nurses. The incorporation of other similar scales, e.g., the Staff Observation Aggression Scale (SOAS) [36], or the Staff Observation Aggression Scale-Revised version [37] may be of further use in examining the concurrent validity and associations of C_MAVAS with these validated scales.

### Limitations of the Study

Though our study defined a four-factor structure for the C_MAVAS, this is not supported by any theoretical framework of perspectives towards patient violence, especially in psychiatric care. The cultural context used for testing this Chinese version much differed from that of the original MAVAS, meaning the use of alternative metrics deserves consideration, as does the nature of staff training and of patient characteristics in any attempt at a comparative study. While the C_MAVAS was directly translated from the original MAVAS, some of the translated items did not necessarily resonate in the contemporary psychiatric care systems and environments. Future studies may consider recruiting nurses from different healthcare settings, both general and psychiatric, so that researchers can explicitly determine whether nurses’ attitudes towards patient violence differ across healthcare settings in diverse populations. Concurrent validity has not been evaluated in this study. Other psychological scales could be incorporated with C_MAVAS to evaluate the concurrent validity in the future. It would be beneficial if a future study could develop a valid, reliable scale able to accurately measure the same construct in these different healthcare contexts in Asia.

## 6. Conclusions

This is the first study to measure the psychometric properties of the C_MAVAS in Hong Kong and is the only Chinese validated and reliable version in empirical research. C_MAVAS demonstrates good content validity, and satisfactory internal consistency and construct validity as a form of measuring mental health nurses’ attitudes towards patient aggressiveness in a psychiatric hospital. The researchers hope to issue a shorter version of the C_MAVAS that can accurately measure nurses’ and patients’ views towards patient aggression in healthcare settings in the future. 

## Figures and Tables

**Table 1:** Demographic profile and occupation-related characteristics of participants (*N* = 262).

Category			*N*	%
Gender				
	Male		102	38.9
	Female		160	61.1
Age group				
	<24		14	5.4
	25–29		38	14.6
	30–39		54	20.7
	40–50		92	35.2
	51–55		48	18.4
	>55		15	5.7
Marital status				
	Single		83	31.8
	Married/Cohabitating		168	64.4
	Divorced/Widowed		10	3.9
Education level				
	Non-degree		68	26.4
	Bachelor Degree		113	44.0
	Master Degree or above		76	29.6
Job position				
	Enrolled Nurse (Psychiatric)		53	20.2
	Registered Nurse (Psychiatric)		131	50.0
	Advanced Practice Nurse (Psychiatric)		50	19.1
	Nurse Officer (Psychiatric)		8	3.1
	Ward Manager (Psychiatric)		16	6.1
	Nurse Consultant (Psychiatric)		2	0.8
	Other		2	0.8
Years of experience in current position				
	0–5 Years		139	53.3
	6–10 Years		56	21.5
	11–15 Years		15	5.7
	16–20 Years		23	8.8
	>20 Years		28	10.7
Years of working in psychiatric hospital				
		0–5 Years	68	26.6
		6–10 Years	30	11.7
		11–15 Years	20	7.8
		16–20 Years	48	18.8
		>20 Years	90	35.2

Percentages may not add up to 100% due to rounding.

**Table 2:** Participants’ experience of WPV in the past 12 months (*N* = 262).

Category		*N*	%
Experience in management of patients’ violence and aggression in the past 12 months			
	Yes	210	80.2
	No	52	19.8
Experience of verbal abuse			
	Yes	199	79.3
	No	52	20.7
Experience of physical assault			
	Yes	166	66.1
	No	85	33.9
Experience of sexual assault			
	Yes	36	14.3
	No	215	85.7
Frequency of being verbally abused by patients in the past 12 months			
	0–5 Times	171	65.3
	6–10 Times	29	11.1
	11–15 Times	13	5.0
	16–20 Times	4	1.5
	>20 Times	45	17.2
Frequency of being physical assaulted by patients in the past 12 months			
	0–5 Times	226	86.9
	6–10 Times	19	7.3
	11–15 Times	8	3.1
	16–20 Times	2	0.8
	>20 Times	5	1.9
Frequency of being sexual assaulted by patients in the past 12 months			
	0–5 Times	255	98.1
	6–10 Times	3	1.2
	>11 Times	2	0.8
Frequency of being verbally abused by patients’ family members/visitors in the past 12 months			
	0–5 Times	234	89.7
	6–10 Times	14	5.4
	11–15 Times	6	2.3
	16–20 Times	2	0.8
	>20 Times	5	1.9
Frequency of being physical assaulted by patients’ family members/visitors in the past 12 months			
	0–5 Times	259	99.2
	6–10 Times	1	0.4
	11–15 Times	1	0.4
Frequency of being sexual assaulted by patients’ family members/visitors in the past 12 months			
	0–5 Times	258	99.2
	6–10 Times	2	0.8
Injury due to patients’ or their family members/visitors’ violence and aggression in the past 12 months			
	Yes	36	14.0
	No	222	86.0
Abrasions			
	Yes	25	61.0
	No	16	39.0
Bruises			
	Yes	22	53.7
	No	19	46.3
Swelling			
	Yes	13	31.7
	No	28	68.3
Hematoma			
	Yes	0	0.0
	No	41	100.0
Other type of injury			
	Yes	3	7.3
	No	38	92.7
Reported aggression/violent incidents			
	Yes	150	64.7
	No	82	35.3
Attended training course on management of violence in the past 5 years			
	Yes	252	96.6
	No	9	3.4
Violent/aggressive incidents can be prevented in your workplace			
	Yes	202	77.1
	No	60	22.9
Feeling safe in workplace			
	Yes	227	86.6
	No	35	13.4

Percentages may not add up to 100% due to rounding.

**Table 3:** Varimax rotation results of C_MAVAS and factor loading of the four-component factor analysis (*N* = 262).

Items	*N*	Mean ± SD	Component			
			1	2	3	4
Improved one to one relationships between staff and patients can reduce the incidence of patient aggression and violence.	262	2.18 ± 0.67	0.672			
The use of de-escalation is successful in preventing violence.	261	2.22 ± 0.63	0.639			
The use of negotiation could be used more effectively when managing aggression and violence.	262	2.22 ± 0.65	0.607			
Aggressive patients will calm down automatically if left alone.	262	2.65 ± 0.70	0.428			
Patient aggression could be handled more effectively on this ward.	255	2.70 ± 0.77	0.422			
If the physical environment were different, patients would be less aggressive.	261	2.61 ± 0.69	0.400			
It is largely situations that contribute towards the expression of aggression by patients.	262	2.73 ± 0.70	0.310			
Physical restraint is sometimes used more than necessary.	262	3.42 ± 0.95		0.633		
Alternatives to the use of containment and sedation to manage patient violence could be used more frequently.	262	2.98 ± 0.82		0.599		
Seclusion is sometimes used more than necessary.	260	3.14 ± 0.80		0.589		
The practice of secluding violent patients should be discontinued.	262	3.71 ± 0.73		0.531		
Expressions of aggression do not always require staff intervention.	262	3.54 ± 0.88		0.423		
Restrictive care environments can contribute towards patient aggression and violence.	262	2.84 ± 0.79		0.378		
Prescribed medication can in some instances lead to patient aggression and violence.	262	3.04 ± 0.87		0.335		
Poor communication between staff and patients leads to patient aggression.	262	3.18 ± 0.89			0.733	
Patients commonly become aggressive because staff do not listen to them.	262	3.78 ± 0.87			0.708	
Other people make patients aggressive or violent.	262	2.64 ± 0.79			0.626	
Patients are aggressive because of the environment they are in.	262	2.62 ± 0.91			0.520	
Patients are aggressive because they are ill.	260	2.76 ± 0.81				
All patients are verbally abusive.	262	3.65 ± 1.06				0.617
Patients who are violent are often restrained for their own safety.	261	2.48 ± 0.89				0.453
It is difficult to prevent patients from becoming violent or aggressive.	261	3.05 ± 0.98				0.435
There appear to be types of patients who frequently become aggressive towards staff.	262	2.24 ± 0.73				0.432
Different approaches are used on this ward to manage patient aggression and violence.	258	2.11 ± 0.66				0.415
Medication is a valuable approach for treating aggressive and violent behaviour.	262	2.04 ± 0.68				0.411
When a patient is violent, seclusion is one of the most effective approaches to use.	262	2.33 ± 0.73				0.360
Patients who are aggressive towards staff should try to control their feelings.	261	2.16 ± 0.63	0.313			0.338
% of variance explained			14.87	8.33	6.84	5.93

Note: Factor loadings < 0.30 to each component were omitted/deleted.
